# Moving towards a Network of Autonomous UAS Atmospheric Profiling Stations for Observations in the Earth’s Lower Atmosphere: The 3D Mesonet Concept

**DOI:** 10.3390/s19122720

**Published:** 2019-06-17

**Authors:** Phillip B. Chilson, Tyler M. Bell, Keith A. Brewster, Gustavo Britto Hupsel de Azevedo, Frederick H. Carr, Kenneth Carson, William Doyle, Christopher A. Fiebrich, Brian R. Greene, James L. Grimsley, Sai Teja Kanneganti, Joshua Martin, Andrew Moore, Robert D. Palmer, Elizabeth A. Pillar-Little, Jorge L. Salazar-Cerreno, Antonio R. Segales, Mark E. Weber, Mark Yeary, Kelvin K. Droegemeier

**Affiliations:** 1 School of Meteorology, University of Oklahoma, Norman, OK 73072, USA; tyler.bell@ou.edu (T.M.B.); kbrewster@ou.edu (K.A.B.); fcarr@ou.edu (F.H.C.); chris@mesonet.org (C.A.F.); brian.greene@ou.edu (B.R.G.); joshua.martin@ou.edu (J.M.); andrew.d.moore-1@ou.edu (A.M.); rpalmer@ou.edu (R.D.P.); epillarlittle@ou.edu (E.A.P.-L.); kkd@ou.edu (K.K.D.); 2 Center for Autonomous Sensing and Sampling, University of Oklahoma, Norman, OK 73072, USA; gust@ou.edu (G.B.H.d.A.); 5@ou.edu (W.D.); kannegantisaiteja@ou.edu (S.T.K.); salazar@ou.edu (J.L.S.-C.); tony.segales@ou.edu (A.R.S.); 3 Advanced Radar Research Center, University of Oklahoma, Norman, OK 73019, USA; yeary@ou.edu; 4 Center for Analysis and Prediction of Storms, University of Oklahoma, Norman, OK 73072, USA; 5 School of Electrical and Computer Engineering, University of Oklahoma, Norman, OK 73019, USA; 6 School of Aviation Studies, University of Oklahoma, Norman, OK 73072, USA; kencarson@ou.edu; 7 Oklahoma Mesonet, Oklahoma Climatological Survey, University of Oklahoma, Norman, OK 73072, USA; 8 Advanced Technology Initiatives, Choctaw Nation of Oklahoma, Durant, OK 74701, USA; jgrimsley@choctawnation.com; 9 School of Computer Science, University of Oklahoma, Norman, OK 73019, USA; 10 Cooperative Institute for Mesoscale Meteorological Studies, University of Oklahoma, Norman, OK 73072, USA; markw@ou.edu

**Keywords:** atmospheric boundary layer, meteorology, forecasting, risk mitigation, sensor integration, unmanned aerial systems

## Abstract

The deployment of small unmanned aircraft systems (UAS) to collect routine in situ vertical profiles of the thermodynamic and kinematic state of the atmosphere in conjunction with other weather observations could significantly improve weather forecasting skill and resolution. High-resolution vertical measurements of pressure, temperature, humidity, wind speed and wind direction are critical to the understanding of atmospheric boundary layer processes integral to air–surface (land, ocean and sea ice) exchanges of energy, momentum, and moisture; how these are affected by climate variability; and how they impact weather forecasts and air quality simulations. We explore the potential value of collecting coordinated atmospheric profiles at fixed surface observing sites at designated times using instrumented UAS. We refer to such a network of autonomous weather UAS designed for atmospheric profiling and capable of operating in most weather conditions as a 3D Mesonet. We outline some of the fundamental and high-impact science questions and sampling needs driving the development of the 3D Mesonet and offer an overview of the general concept of operations. Preliminary measurements from profiling UAS are presented and we discuss how measurements from an operational network could be realized to better characterize the atmospheric boundary layer, improve weather forecasts, and help to identify threats of severe weather.

## 1. Introduction

Dramatic, high-impact weather events, such as severe thunderstorms with hail and wind, tornadoes, excessive rainfall and flooding, tropical storms, ice storms, heavy snowstorms, and blizzards have an impact of billions of dollars per year on the economy of the United States [1,2]. Additionally, several industries in the U.S. are quite weather sensitive, including agriculture, transportation and electric power generation and management. Definitive links are being made between climate change and the impact it is having on the occurrence of dramatic weather events [3]. To mitigate deleterious impacts on society and its infrastructure, it is imperative that we develop innovative means of monitoring and modeling the Earth’s atmosphere. To achieve this end, we require better observations of the lower atmosphere and an effective means of incorporating these measurements into numerical weather predictions (NWP). That is, the availability of quality atmospheric observations is critical to our ability to monitor meteorological conditions and accurately forecast the weather.

Regarding atmospheric observations, a long-desired component to U.S. operational observing systems is the ability to measure vertical profiles of wind, temperature, and moisture in the lower troposphere at high spatial and temporal resolution. These so-called sounding or profiling data can be used to assess regions of thermal stratification and the degree of atmospheric static and dynamic stability, which play a role in convection initiation and maintenance of storms entering an area. This need is reflected in several recent studies, some of which provide explicit recommendations to collect more observations within the atmospheric boundary layer (ABL) in general, with a focus on vertical sampling (profiling) in particular [4,5,6,7,8,9]. These reports emphatically state that our currently available observing systems are not capable of providing adequately detailed profiles of temperature, moisture, and winds within the ABL.

**Figure 1:**
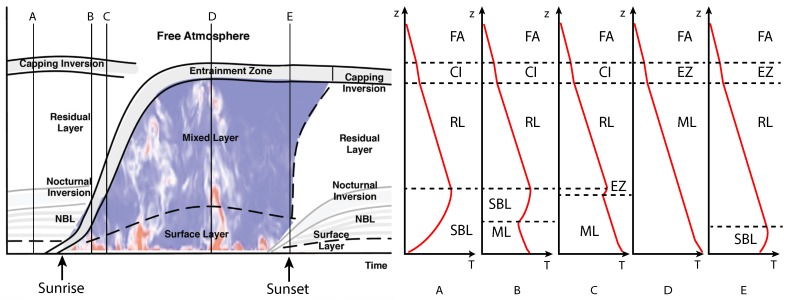
Schematic depicting the idealized structure of the ABL (**left**) during one diurnal cycle under quiescent conditions. Vertical profiles of the temperature at five particular times (denoted as A–E) are presented to the (**right**). In this cloud-free example, the structure of the ABL is primarily driven by thermal forcing produced by insolation.

Overall, processes in the ABL can vary dramatically over a single diurnal cycle, as depicted in Figure 1. Although this conceptual model of the ABL is idealized, it helps to illuminate several common features of the ABL structure: mixed layer (ML), capping inversion (CI), the stable boundary layer (SBL), entrainment zone (EZ), the residual layer (RL), and so forth. Above the ABL is the free atmosphere (FA). The temperature profile corresponding to five particular “snap shots” of this idealized ABL diurnal cycle are depicted and labeled as A–E. Time (A) corresponds to nocturnal conditions characterized by a stable boundary layer near the surface with a well mixed residual layer above. Shortly after sunrise, the ABL begins to transition as depicted at times (B) and (C). The mixed layer is forming near the surface and as it continues to grow, the stable boundary layer is lifted and compressed until it later forms the entrainment zone. During mid day, the ABL is largely characterized as a mixed layer as shown at time (D). At sunset, surface cooling begins to occur, which in turn sets up the development of the stable boundary layer again, as shown at time (E). To fully characterize the ABL, measurements of the state parameters in each of these regions are needed, preferably with adequate temporal resolution to fully capture evolution of the height of the ABL and structures within the ABL.

The ABL generally provides the moisture, instability, low-level wind shear, and forcing necessary for the formation of severe storms with attendant tornadoes, hail, lightning, and hazardous winds. Forecasters regularly look at moisture advection and moisture gradient patterns when considering the potential for convection initiation [10,11].Within the ABL resides the storm-generated outflows that can regulate the strength and longevity of severe storms or even trigger new storms. Knowledge of these conditions is the key to improving predictions of severe weather events. The problem is that ABL properties are highly variable on mesoscale time and space scales, which are virtually undetected by current operational observing systems.

Vertical shear in the near-ground layer can also play a significant role in severe weather formation and is an important criterion when distinguishing tornadic from nontornadic supercells. Traditional observational data, such as those provided by wind profiling radars do not offer adequate vertical resolution to resolve the shear (e.g., [12]). The most noticeable difference between nontornadic and tornadic cases is in the lower-tropospheric wind profile, specifically, the orientation of the 0–500 m shear vector with respect to the storm-relative inflow. This implies that the tornadic cases have much more streamwise horizontal vorticity in the lowest 500 m AGL [13]. Traditional standards used for radiosonde data processing do not capture features in the lowest 500 m well, partly on account of the pendulum effect [14]. Therefore, there is a need to sample the ABL near the surface with finer vertical resolution.

To address the need for more measurements in the ABL, advance several key recommendations listed in the latest NASA Strategic Plan [15], and fulfill the mandates put before NOAA in the 2017 Weather Research and Forecasting Innovation Act, we must challenge ourselves to develop observing and modeling systems that transcend conventional methods. One solution is to use remote sensing technologies to sample the thermodynamic and kinematic properties of the lower atmosphere. Using funds from a COST action initiative, which allows European Union researchers to form interdisciplinary teams to address pressing scientific and societal challenges, investigations are underway to assess the potential impact of ground-based profiling on weather forecasting [16]. This initiative focuses on the combined use of ceilometers (referred to in the paper as ALC or automatic low-power backscatter lidars/ceilometers), Doppler wind lidars, and microwave radiometers to retrieve measurements of temperature, humidity, aerosols, and wind. It has been demonstrated by Manninen et al. [17] that Doppler wind lidars can be used to identify sources of turbulent mixing. There have been many ABL field studies in the last decades, which have relied on multiple ground-based remote sensing technology to observe the lower atmosphere. Some recent examples include those discussed in Lothon et al. [18], Klein et al. [19], Fernando et al. [20] to study the ABL. Despite encouraging results, this approach has several notable disadvantages, namely, the need for multiple remote sensing systems and the cost of purchasing the equipment; power requirements; lack of data availability within fog, clouds, and precipitation; and the need to rely on indirect approaches to retrieve the desired atmospheric parameters. Moreover, wind estimates from scanning Doppler systems such as Doppler wind lidars, radar wind profilers, and sodars are all potentially affected by complex terrain (e.g., [20,21]).

Another emerging technology which could have a dramatic impact on observations of the lower atmosphere is unmanned aircraft systems (UAS). It has been suggested that such profiling could be achieved by small UAS assuming that autonomous flights extend at least through the depth of the boundary layer [22]. As outlined below, instrumented UAS are expected to provide inexpensive, accurate, and controlled observations of the lower atmosphere. The advent of weather-observing UAS (WxUAS) would complement other observing systems, such as rawinsondes, towers, satellite-based remote sensors, and active and passive ground-based remote sensors. Here, we focus on how WxUAS data would complement measurements from networks of surface-based, in situ observing stations, known as mesonets.

In recent decades, numerous states have established mesonet networks to aid decision making across various sectors ranging from emergency management to agriculture to weather forecasting to transportation [23,24]. In general, mesonets aim to provide multi-purpose, high-quality, real-time observations. Additionally, they provide tailored outreach (typically to the K-12, university, public safety, and agriculture communities) to expand the utility of the observations. Across the U.S., there are currently 27 statewide mesonets [23]. These networks range in size from less than 10 stations to over 175 stations, but each has a goal of providing high resolution observations to support mesoscale weather and climate monitoring. Typically, mesonets include sensors mounted on or below a 10 m tower to sample air temperature, relative humidity, winds, solar radiation, precipitation, pressure, soil temperature, and soil moisture. The New York Mesonet additionally has profiling capability at 17 sites [25]. This is achieved using scanning Doppler lidars (3D wind) and microwave radiometers (temperature and humidity). We should also mention that the West Texas Mesonet has profiling capability at a single site through the operation of a radar wind profiler and a sodar [26].

**Figure 2:**
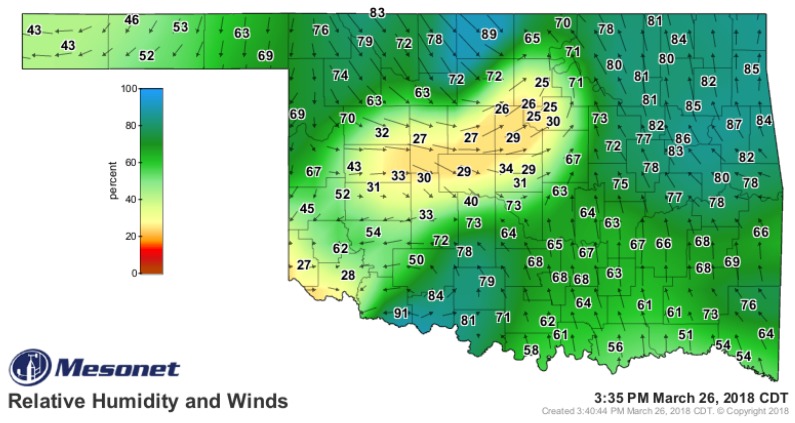
Mesonet station plot of relative humidity (%) and wind field vectors across Oklahoma during the afternoon of 26 March 2018.

In Figure 2, we present an example of the surface mesoscale wind and humidity fields on the afternoon of 26 March 2018 as recorded by the Oklahoma Mesonet [27,28]. A combination of varying insolation across the state and the action of a cold front, dryline, and upper-level jet resulted in significant county-by-county variability. The implications of such features on wildfire risk (including likelihood for initiation, behavior, and smoke dispersion), convective initiation, and moisture and momentum fluxes are immense, yet inadequately understood without additional measurements of the ABL.

Mesonets can provide valuable information on the spatiotemporal structure and development of events such as the one depicted in Figure 2; however, measurements are mostly limited to the surface layer. The vertical structure remains under sampled. Therefore, forecasters rely on statistically-based parameterization schemes [29] and basic conceptual models to envision processes acting in the vertical dimension. Providing researchers and forecasters with data that allow them to monitor the changing 3D wind, temperature, and moisture patterns would yield considerable benefits. For example, subtle changes in the strength of the capping inversion can have a profound impact on the probability of convective initiation and the chance that storms may become severe. Further, these data can be used to initialize mesoscale and thunderstorm-scale NWP models. Knowing if, when, and where the cap might “break” is paramount to anticipating where convection could be initiated. Current parameterization schemes struggle to provide adequate information on the strength of the capping inversion, mostly due to lack of observational data.

Here, we explore the prospects of extending the mesonet concept beyond the surface by including the capacity to sample the vertical structure of the Earth’s atmosphere through the use of WxUAS. That is, 2D surface observations from tower based sensors would be complemented by profiling measurements within the ABL and lower free troposphere from instrumented unmanned aerial vehicles (UAVs) launched from a network of ground stations capable of supporting these operations. As a point of clarification, we will use the terms UAV or WxUAV when referring to actual aircraft and UAS or WxUAS in the context of the complete system, including the aircraft, ground station, and other components associated with operations. The WxUAS would add a third spatial dimension to the sampling strategy of conventional mesonets and, as such, the proposed concept is referred to here as a *3D Mesonet*. This framework would allow us to fill data gaps in the ABL that conventional instrumentation cannot easily or feasibly provide and facilitate the detection of complex mesoscale features embedded in weather systems. As we have noted, the New York Mesonet has implemented ground-based remote sensing platforms to obtain profiling data at select sites. However, we see WxUAS as being a less expensive alternative to this approach, albeit with several logistical challenges that need to be addressed.

In the following sections, we begin with a general overview of the 3D Mesonet concept and its potential impact on weather forecasting. Then, we discuss the development, calibration, and validation of a WxUAV designed for the needs of the 3D Mesonet along with other components needed for operations and risk mitigation. Next, we present the data visualization system and some preliminary results that highlight the unique capabilities the WxUAS has at profiling the ABL. Finally, we will outline the anticipated trajectory of the system along with future work that would be needed to transform the 3D Mesonet concept from a fundamental research question to an operational system.

## 2. Overview of the 3D Mesonet Concept

### 2.1. Growth of WxUAS

There has been rapid development in WxUAS technology and its integration into meteorological research for atmospheric boundary layer studies [30,31,32]. Taking its cues from manned aircraft operations, many of the initial studies with WxUAS were conducted using fixed-wing aircraft. An overview paper of these platforms can be found in Elston et al. [33]. More recently, however, rotary-wing vehicles are playing a prominent role in WxUAS research (e.g., [34,35,36]). There have been notable strides towards the integration of rotary-wing WxUAS into atmospheric studies; however, the UAS community has yet to adopt standards or recommendations as to the types of rotary-wing aircraft or sensor suites best suited for particular sampling needs. Many research groups are using commercially available rotary-wing UAV with modifications of some sort to achieve their respective measurement goals. Modifications could include adding custom sensor packages to the airframe, making alterations to the autopilot system, adding additional telemetry capability for real-time communication with sensors, or so forth.

There are very few rotary-wing WxUAS commercially available which offer all measurement and telemetry capabilities envisioned for autonomous operations. One exception is the collection of Meteodrones developed by Meteomatics, AG in Switzerland. This line of WxUAS has been specifically engineered to autonomously collect and report atmospheric profiles of pressure, temperature, humidity, wind speed, and wind direction. Additionally, Meteomatics has recently introduced a charging box, which can be used with the Meteodrones. The University of Oklahoma (OU) CopterSonde, which is briefly described in Section 3 has a similar design philosophy as the Meteodrone. Both systems were used during the Environmental Profiling and Initiation of Convection (EPIC) experiment [37] conducted in Oklahoma. Since EPIC, both OU and Meteomatics have made significant improvements to their systems. Notable improvements to the CopterSonde are documented in Greene et al. [38], Greene et al. [39], and Barbieri et al. [40].

### 2.2. Conceptual Framework of the 3D Mesonet

Our concept of a 3D Mesonet consists of several fundamental components: (1) a network of tower-based surface observing stations distributed over a designated surface area; (2) a potentially less spatially dense yet complementary network of ground stations from which small WxUAVs can be launched, recovered, and reused; (3) the ability to operate unattended and with minimal human interaction in diverse weather conditions; (4) a method of monitoring the airspace in which the WxUAVs are operating and mitigating the risk of potential air collisions between WxUAVs and manned aircraft and between WxUAVs and other UAVs (deconfliction); (5) a robust and reliable methodology for communicating operational critical commands between the WxUAV and the ground station; and (6) a robust and reliable method of communicating data from all sensors to a physical and/or cloud-based command center. Since the focus is on profile data, the WxUAV would execute a vertical ascent and descent. The proposed modular system will allow for customization, upgrade, and in-field replacement of sensor packages as desired. Such a system would facilitate off-site maintenance and calibration and would provide the ability to add new sensors as they are developed, or as new requirements are identified. The small WxUAV must be capable of handling the weight of all sensor packages and have lighting, communication, and aircraft avoidance systems necessary to meet existing or future FAA regulations. The system must be able to operate unattended or with remote pilots at such time that FAA regulations allow.

**Figure 3:**
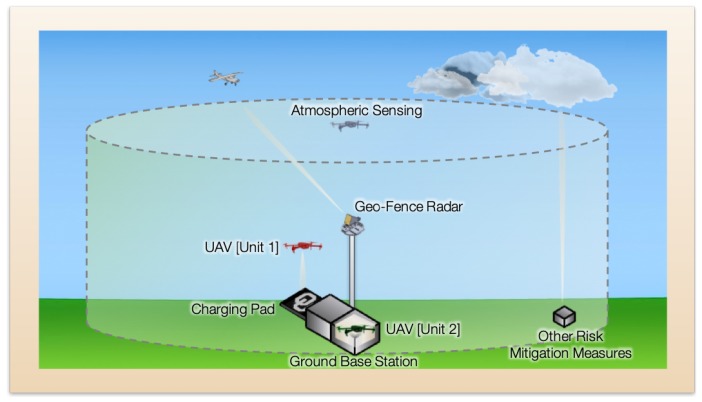
Conceptual depiction of a 3D Mesonet site. The dashed cylindrical boundary represents the extent of the geofenced airspace.

Regulations regarding access to and operations in the National Airspace System (NAS) are continuously evolving, so it is difficult to anticipate the level of risk mitigation required to satisfy the FAA. We propose to designate a cylindrical “geofence” within the NAS, with the center of the cylinder corresponding to the vertical flight path of the WxUAV (Figure 3). If an aircraft enters the geofenced volume, then instructions are communicated to the WxUAV for evasive actions to deconflict the airspace. Based on current assessments of the regulations, our design includes a radar system to detect the presence of small manned aircraft coupled with the ability to monitor signals from Automatic Dependent Surveillance-Broadcast (ADS-B) equipped aircraft. A sophisticated software package will be needed to translate inputs from the radar and ADS-B system into appropriate flight commands for the WxUAV to deconflict the airspace.

To facilitate unattended and autonomous operations, several other components are required for the 3D Mesonet station. For example, some form of charging pad is needed to charge the batteries in the WxUAV. A housing or shelter is also necessary to protect the WxUAV from the elements between flights. It will also be necessary to conduct diagnostics checks of the WxUAS before, during, and after flights. These functions will be facilitated through a ground control station, which will serve as the central hub for the WxUAS. It will manage communications and control as well as video monitoring of the WxUAV between and during flights. The ground control station and other elements of a 3D Mesonet station are provided below. A conceptual illustration is provided in Figure 3.

A prototype 3D Mesonet WxUAS station is being developed, constructed, and tested at OU under the direction of the university’s Center for Autonomous Sensing and Sampling (CASS). CASS is an interdisciplinary center with the primary focus of helping to develop a better understanding of the environment in which we live through the assistance of autonomous systems. The development site for the prototype is the OU Kessler Atmospheric and Ecological Field Station (KAEFS), which is located about 28 km south-southwest of the main OU campus. KAEFS hosts a wide variety of research thrusts and instrumentation. Among these is one of towers in the Oklahoma Mesonet—the Washington (WASH) site. CASS conducts much of its WxUAS research at KAEFS and the prototype is co-located with the WASH Mesonet tower. As we discuss below, several of the requisite components of a 3D Mesonet WxUAS station are already being tested and integrated at KAEFS.

### 2.3. Impact of Data on Weather Forecasting

Before building out a fully functioning 3D Mesonet, there are several questions that can be explored regarding how the individual WxUAS stations should be deployed and operated and what potential impact one expects. For example, what would be the optimal horizontal spacing of the WxUAS? To what height should the WxUAV ascend? At what frequency should the WxUAV be operated? There is still much work to be done, but we have completed a preliminary study to examine the potential improvement that a WxUAS network could have on storm-scale NWP using an Observation System Simulation Experiment (OSSE) approach. An OSSE was performed over the state of Oklahoma in which it was assumed that a WxUAV could be launched from 110 Oklahoma Mesonet [28] stations hourly, fly vertically to an assigned maximum altitude, and return to its charging station [41]. This approach would provide soundings at a roughly 35 km horizontal resolution. A case study focusing on convective initiation was chosen as a compromise between a fair-weather day and one with extensive ongoing convection. The OU Advanced Regional Prediction System (ARPS) [42] model provided a nature run at high (1 km) resolution, while the control run and OSSE experiments were done with the WRF-ARW (Weather Research and Forecasting–Advanced Research WRF) model at 3 km. To serve as a proxy for all the data from dozens of observing systems already assimilated into larger-scale operational models, the nature run data volume was sampled on a one-degree latitude–longitude grid and inserted into the control run at 3-h intervals. Simulated hourly WxUAV-measured temperature, moisture and wind data, with expected errors, were then added in a 6-h data assimilation (DA) window, followed by 12-h forecasts. The analyses and forecasts were examined to assess the added value of WxUAV data. Tests were run to measure the impact of varying the maximum WxUAV altitude and the spatial density of WxUAS observations.

**Figure 4:**
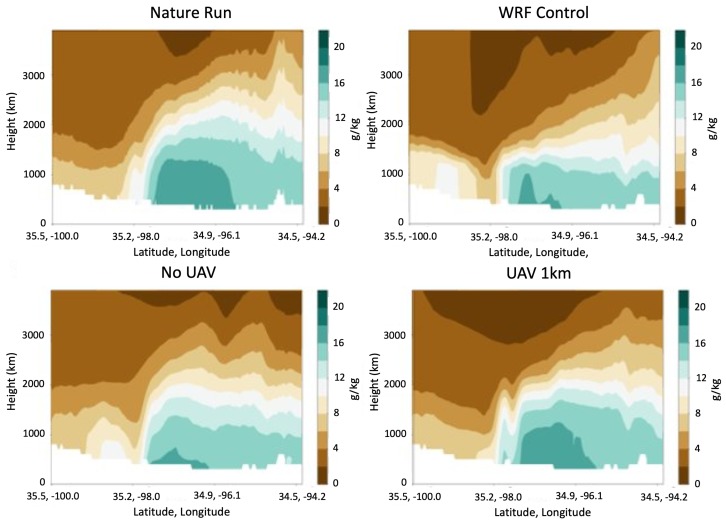
Cross section plots of mixing ratio for the Nature Run (NR), WRF Control, No WxUAV, WxUAV up to 1 km, WRF analyses valid at 1800 UTC 20 May 2013.

**Figure 5:**
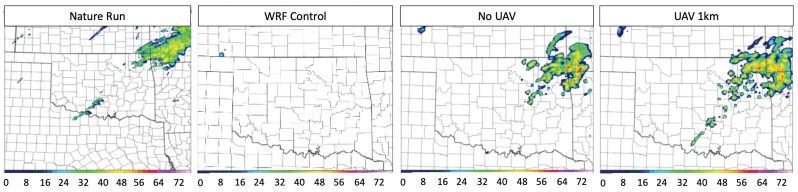
Comparison between modeled composite radar reflectivity (dBZ) between the Nature Run (NR), WRF Control, No WxUAV, WxUAV up to 1 km at 1900 UTC on 20 May 2013.

Regarding the question of maximum WxUAV altitude, four experiments were run comparing no WxUAV flights and flights up to 400 ft, 1 km, 2 km, and 3 km AGL. In the test case storm initiation was impacted by a dry line extending through Oklahoma and progressing eastward. Initial results clearly show an improved boundary layer structure and subsequent convective initiation location and timing when WxUAV data are used. Figure 4 includes cross sections of specific humidity for the ARPS nature run; the WRF control run with no DA; the WRF run with DA but no WxUAV data; and the WRF run including WxUAV data up to 1 km. It was determined that the addition of flights up to 1 km was adequate to reliably resolve the vertical structure of the humidity field in the ABL. Moreover, when focusing on storms that initiated in south-central Oklahoma, which evolved into the most severe tornadic storms later that day, we found that including WxUAV flights up to 3 km AGL produced the best results; however, the 1 km WxUAV DA runs also showed success in reproducing convection initiation in southwest Oklahoma, as shown in Figure 5.

Considering network density, experiments were also conducted involving simulated WxUAS stations located at 110, 75, 50, 25, and 10 Mesonet sites. Simulated WxUAV flights went up to a height of 1-km AGL. It was found that, while a WxUAS network consisting of 110 3D Mesonet stations produced the best results, the convective initiation forecast was improved with only 75 stations. It is worth noting that results from the study suggest that even a 25-station network could provide benefit to boundary layer analyses and short term convective forecasts. In summary, although sensitivities to the quality of the moisture analysis are noted, the results suggest that a real-world deployment of automated WxUAVs could have a positive impact on atmospheric analyses and short-term NWP of convective initiation. Again, complete results can be found in Moore [41] and a forthcoming paper.

## 3. Platform and Sensor Development

The general concept of operations for the 3D Mesonet places certain design criteria on the WxUAV to be used. Flights should be conducted autonomously or semi-autonomously with minimal human interactions. Moreover, it should consist of a vertical take-off and landing (VTOL) aircraft for the sake of docking and charging. This can be achieved using a rotary-wing aircraft or a hybrid vehicle capable of operating in a rotary-wing mode for take off and landing and then transitioning into a fixed-wing aircraft as its primary mode for data collection. Here, we present developments of a rotary-wing VTOL vehicle, known as the CopterSonde. It should be noted that the WxUAV described below is still undergoing modifications. Therefore, it should be taken as representative of the aircraft to be used as part of the 3D Mesonet. However, we are also exploring hybrid vehicles, which are under development within CASS and will be presented in forthcoming publications.

**Figure 6:**
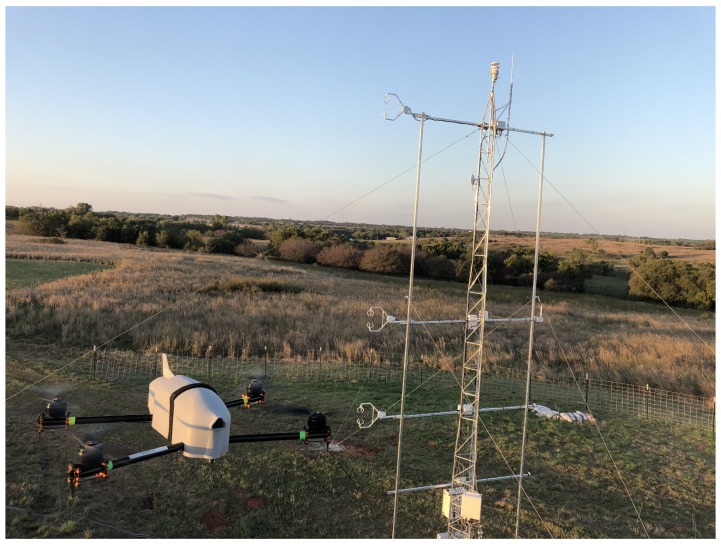
Picture showing the CopterSonde in flight near a meteorological tower.

The CopterSonde series rotary-wing WxUAV was developed in-house by a CASS team of engineers and meteorologists to be both robust and optimized for atmospheric sampling. True to its name, the platforms are able to collect vertical profiles of the atmosphere like a traditional radiosonde. In addition, these platforms provide highly resolved data (<10 m) in the lower atmosphere that are not easily achievable using conventional methods. The CopterSonde (Figure 6) is based on the HQuad500 manufactured by Lynxmotion. It is comprised of carbon fiber plates, aluminum brackets, and carbon fiber tube legs. The rotary-wing craft is electrically powered by a lithium polymer battery pack with a capacity of 6750 mAh, which provides a maximum flight endurance of 25–30 min and can allow the platform to climb to an altitude exceeding 2000 m AGL. It has been operated in Colorado to heights of 3000 m ASL. The propulsion system of the CopterSonde is comprised of four identical brushless motors outfitted with T-style carbon fiber propellers and a 35-A 4-in-1 electronic speed controller. The electronic components are protected from the elements with a custom 3D printed two-piece plastic shell, which was designed to enhance the vehicle’s aerodynamics and allow for easy access to the interior components. Additional information can be found in Greene et al. [39].

All CopterSonde platforms utilize open-source technologies to enable autonomous operation of the vehicle. The first of these is a Pixhawk 2.1 flight controller (ProfiCNC), which is comprised of a rugged and vibrationally isolated microcontroller, a single central processing unit that executes the flight control system and also allows for communication with a large variety of sensors over a wide range of communication protocols (I2C, UART, RS232, etc.) When paired with the Here+ GPS module (ProfiCNC), the Pixhawk is able to utilize Real Time Kinematic (RTK) GPS, which can reduce the uncertainty of the location of the UAS to less than 1 m in both the horizontal and vertical if properly set-up and calibrated. The microcontroller runs the ArduPilot autopilot software suite, which is composed of navigation software on-board the vehicle. The autopilot comes with a ground station software which is used to transmit commands to the UAV and monitor its status over telemetry link. Both software packages are open-source for any developer, which allows to create highly customizable environments/configurations and the addition of code that enables for other sensor integration and special features, such as to dictate the sampling speed of the sensors, flight path of the vehicle, ascent rate, and much more.

The design of the CopterSonde has been driven by meteorological sampling needs coupled with the overall concept of operations and the logistical requirements of unattended operations. Meteorological sampling needs include both the types of measurements to be acquired and the accuracy of those measurements. For the 3D Mesonet concept, we are primarily interested in characterizing the thermodynamic and kinematic state of the lower atmosphere, namely, atmospheric pressure, temperature, and humidity along with the wind speed and direction with *sufficient* temporal and vertical resolution. For reference, we present in Table 1 the variables to be observed using the CopterSonde and their respective target accuracies. To achieve these measurement goals, it has been necessary to carefully explore how to provide adequate aspiration of the sensors, minimize the effects from direct exposure to the sun, and insure that the measurements are not being impacted by affects from the vehicle itself, such as heating. More information on sensor placement on the CopterSonde can be found in Greene et al. [38] and Greene et al. [39]. An intercomparison of thermodynamic and kinematic measurements from various WxUAVs, including the CopterSonde, can be found in Barbieri et al. [40].

The CopterSonde is equipped with three Innovative Sensor Technology HYT 271 humidity sensors, three InterMet Bead Thermistors, and a MS5611 Barometer to measure pressure. The temperature and humidity sensors are located on the front of the vehicle in the 3D printed scoop designed to protect the sensors from solar radiation and serve as the front half of the platform’s protective shell [39]. A ducted fan is placed in the bottom of the scoop and used to aspirate the sensors by pulling air over them at a constant rate. The barometer is located inside of the Pixhawk flight controller. Wind velocity and direction is calculated using an algorithm in the autopilot software, Ardupilot, which uses the pitch, yaw, and roll angle of the platform. Additionally, the rotary-wing craft uses the results of the wind algorithm to keep the vehicle facing into the wind as to allow for sampling of air undisturbed by the propellers.

Wind speed and direction is estimated as follows. The autopilot system has been configured such that the CopterSonde can orient into the direction of the incoming wind. Since the pitch, roll and yaw of the vehicle are measured, wind direction is found from the yaw angle [39]. The wind speed is then calculated from the pitch angle using a method similar to that described by Neumann and Bartholmai [43] and Palomaki et al. [44]. The requisite coefficients for the wind speed calculations are obtained by calibrating the wind retrieval algorithm as the CopterSonde hovers near a meteorological tower.

## 4. Supporting Components of the 3D Mesonet

### 4.1. Ground Control Station

**Figure 7:**
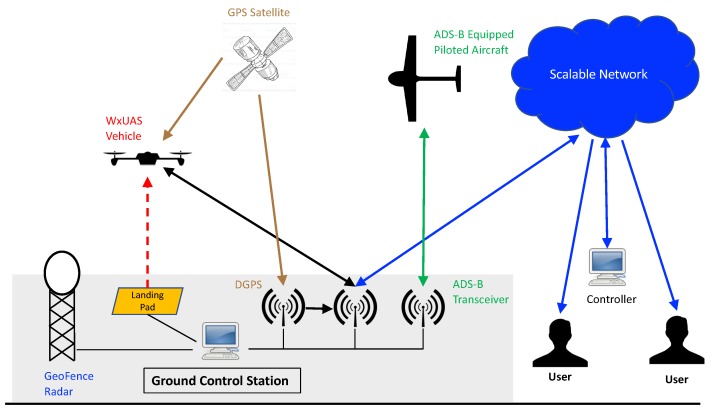
Illustration of how the Ground Control Station is used at a particular 3D Mesonet station to network with the WxUAV, the risk mitigation components, and the rest of the world. The extra DGPS (Differential Global Positioning System) antenna in the GCS is used to improve the accuracy of the estimated position of the WxUAV during flight.

As a whole, the ground control station (GCS) for a single 3D Mesonet unit serves as the primary information conduit between the WxUAV and the rest of the world and is used to coordinate the functionality of the various mission-critical components required for the WxUAS operations. A schematic representation of how the GCS interacts with these components is depicted in Figure 7. The GCS remains in continuous communication with the WxUAV and allows the user to visualize and interpret operational information and data coming from WxUAV in real time. This information can be evaluated at the field site or remotely through a scalable cloud-based network. The data streams can also be used as inputs for various analysis software or assimilated into weather forecast models. The GCS is vital for autonomous and semi-autonomous operations of the WxUAV as it is used to monitor the electrical and mechanical integrity of the WxUAV before, during, and after flight. The GCS monitors local weather conditions from the Mesonet tower it accompanies and assesses whether a flight should be initiated before takeoff or in the the case of an ongoing flight, if it should be terminated. The GCS continuously ingests and processes data coming from the risk mitigation instrumentation associated with the 3D Mesonet station, such as the GeoFence Radar and ADS-B transceiver, to assist in deconflicting the airspace in the case other manned or unmanned aircraft pose a threat of collision with the WxUAV. These risk mitigation instruments are described in more detail below.

### 4.2. Risk Mitigation: GeoFence Radar

**Figure 8:**
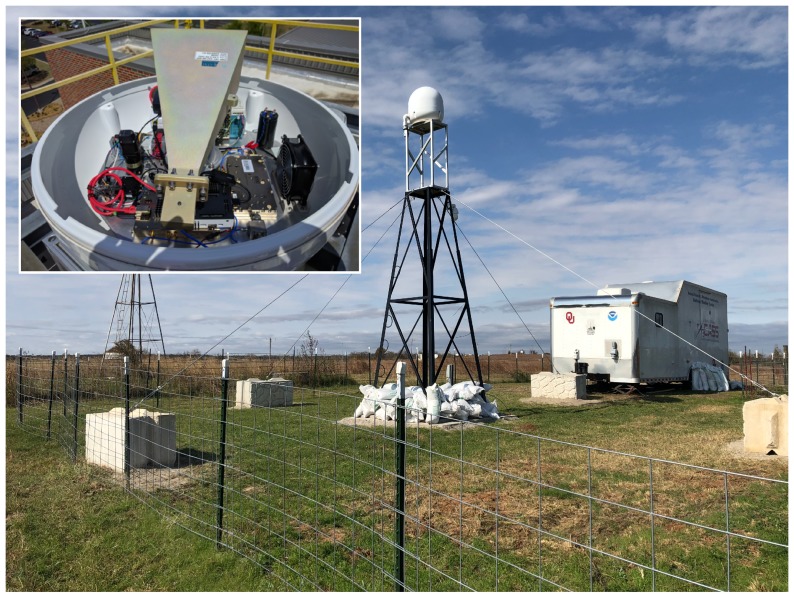
Picture of the GeoFence Radar mounted on a tower at KAEFS with an inset picture showing the radar hardware.

To promote safe operations of the WxUAV in the NAS as part of the 3D Mesonet, we are developing radar technology that supports beyond-visual-line-of-sight (BVLOS) WxUAS operations within a specified (geofenced) airspace volume. Namely, the specific goal is to demonstrate the utility of deploying a low-cost, readily manufactured ground-based detect-and-avoid (GBDAA) radar at the 3D Mesonet. The radar has been designed to detect manned aircraft. A prototyple of the so-called GeoFence Radar has been built in the team’s lab at the Advanced Radar Research Center (ARRC) at the University of Oklahoma. The GeoFence Radar is capable of detecting aircraft that enter into the geofenced volume. It has been tested by flying aircraft from the university’s Department of Aviation Studies in specific patterns to confirm radar detections. The radar’s primary specifications are: 5.6 GHz operating frequency, 10–20 MHz of operating bandwidth, 6.3 kW peak transmit power, maximum duty cycle = 10%, range = 5 km, probability of detection within range = 99.9%. As such, the radar scans continuously, aiming for a high probability of detecting targets entering a 5 km range. Power supplies, a processing unit, an antenna, and radio frequency components for both transmitting and receiving signals have been designed for the radar. A picture of the radar deployed at a test site in Oklahoma (KAEFS) is provided in Figure 8.

To detect targets, we implemented an algorithm derived from a traditional Constant False-Alarm Rate (CFAR) detection algorithm. This algorithm uses radar data to estimate random noise levels and distinguish targets from noise. While operating, the GeoFence Radar generates a stream of measurements, each pointed in the current direction of the radar as it spins. Once the radar completes a full circle, the receiver has assembled a Plan Position Indicator (PPI). The second component of the program is our CFAR implementation, which plots detections on an image of the PPI. We were successful in running the program in real time, ideally allowing for immediate visualization of aircraft within the radar’s effective range. The program also saves data from completed PPIs and periodically stores the resulting visualization for later review. We conducted several flight tests. The aircraft target used was a single-engine, four-seater Piper Warrior III, a common aircraft which served as a type specimen for the light aircraft GeoFence Radar was designed to detect. This test consisted of a series of maneuvers that aimed to test the capabilities of the GeoFence Radar. We developed a flight plan which ensured that the radar could view its target at many different altitudes and ranges.

**Figure 9:**
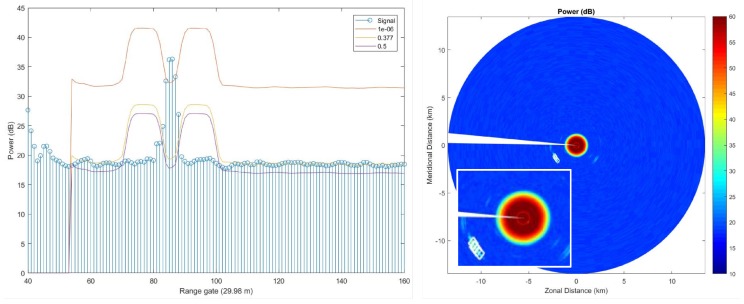
(**left**) Plot of the returned power versus range corresponding to the detection of an aircraft with three different CFAR thresholds. See text for detail. (**right**) A plot showing the returned power for all radials along with detections. The insert is a zoom of the detections.

On 26 February 2018, the flight plan began with the plane approaching the location of the radar from Max Westheimer Airport. The the Piper Warrior III airplane then proceeded to complete two figure-eight patterns of one-mile radius loops. The plane then ascended to 1500 ft (460 m), completed two passes over the radar, and ascended to 5000 ft (1520 m). The final maneuver of the plan was to complete a loop four miles (6.4 km) in radius before returning to the airport. Specific detections are shown in Figure 9. In particular, the graph on the left depicts the behavior of the CFAR algorithm. It shows the returned power for a single radial in the radar scan corresponding to the detection of an aircraft alongside CFAR thresholds from different probability of false alarm ($P_{fa}$) values. The aircraft is located at about range gate 80 and the $P_{fa}=1\times 10^{-6}$ threshold clearly just includes the aircraft. The other thresholds of 0.377 and 0.5 are for significantly higher probabilities of false alarm and would not be used for actual detection. The figure on the right depicts a set of detections in the lower-left quadrant. The right wedge in the left of the figure depicts strong reflections from a nearby water tower.

### 4.3. Risk Mitigation: Automatic Dependent Surveillance-Broadcast (ADS-B)

The ADS-B system is widely used surveillance technology for tracking the location, speed, and heading of aircraft. An ADS-B equipped aircraft determines its position using satellites and on-board calculations are made to estimate other navigational information. The information is broadcast using ADS-B out. Air traffic controllers and other pilots can also monitor the tracking information of other ADS-B equipped aircraft using ADS-B in. This helps to deconflict the NAS and allow individual pilots to exercise self separation. The incorporation of ADS-B technology is part of the FAA Next Generation Air Transportation System (NextGen). All aircraft operating in controlled airspace in the US will be required to have ADS-B out capability by 2020.

Although there are several commercially available ADS-B hardware/software packages that allow aircraft monitoring, we have opted to develop our own system of registering and visualizing flight tracks using data from an ADS-B receiver. This provides the versatility to not only track an aircraft but also predict its future location based on past trajectory data. The forecast algorithm is still in development, but when completed, should provide additional warning of incoming aircraft and assist in the decision process to abort a WxUAV flight or instigate evasive maneuvers. The hardware we have selected to receive ADS-B signals is the PingStation from uAvionix. This receiver is a dual band networkable receiver with Power over Ethernet (POE). The PingStation detects ADS-B equipped aircraft within a 240-km (150-mile) radius. The PingStation is robust enough to be used in harsh environmental conditions and small enough to be used as a mobile asset for roaming operations. It uses an Ethernet connection and communicates using JSON UDP. Specifications for the PingStation are provided in Table 2.

**Figure 10:**
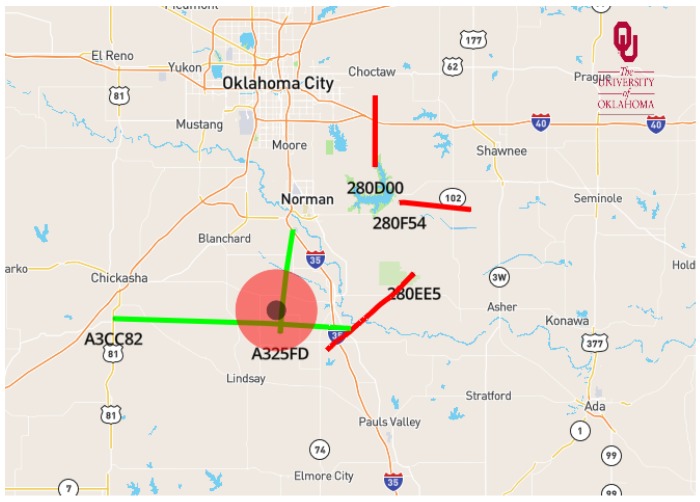
Screen shot of air traffic detection and tracking using the ADS-B software being developed for the 3D Mesonet. In the image, the red circle denotes the boundaries of the geofenced air space designated during operations. Red and green flight tracks indicate aircraft that are below and above 5000 ft (1525 m), respectively.

In the current configuration of our flight tracking software, data from aircraft are obtained using the PingStation and then sent to a processing computer by configuring Dynamic Host Configuration Protocol (DHCP) connection between the receiver and processing computer. A visualization software was developed in Python v3.6 and utilizes Mapbox, which provides custom online maps for websites and applications. This has allowed us to build our aircraft visualization applications, which are flexible and portable. Data received from the PingStation are used to view air traffic in a web browser, which updates air traffic on the map every two seconds. A more detailed discussion can be found in Kanneganti et al. [45]. An example of how flight tracks are displayed through the software is given in Figure 10. The red circle indicates the designated horizontal extent of the geofenced air space during WxUAV operations. The red and green lines show flight trajectories of several aircraft, with their respective ICAO (International Civil Aviation Organization) address indicated at the point of the current location. Here, green lines indicate that the aircraft is above 5000 ft (1525 m) and deemed to be not a risk. Flight trajectories below 5000 ft are marked as red. The radius of the geofence cylinder and the risk/no risk threshold height can be set in the software.

## 5. Data Processing, Distribution, and Visualization

### 5.1. Data Processing, Distribution, and Visualization

**Figure 11:**
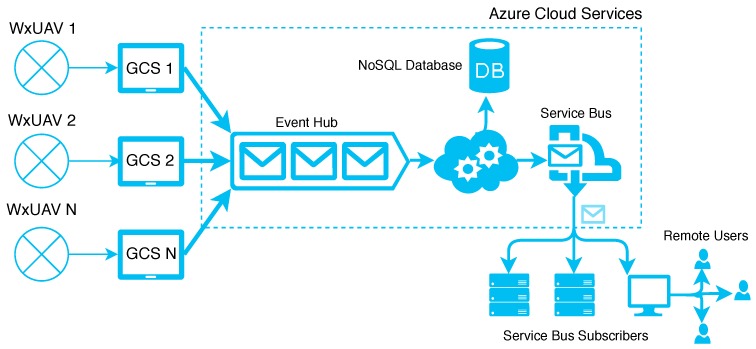
Diagram showing the flow of data from the WxUAV, through the GCS, to the cloud, and then to subscribers and remote users.

Using the current and planned architecture, the work load of data processing, distribution, and visualization can be achieved on the GCS, in the cloud, and on remote servers. We provide an example of the data flow from the WxUAV (CopterSonde) to remote users in Figure 11. The concept of operations is designed to allow flexible allocation of computing resources based on need. We can harness the power of Cloud-based resources; however, operations and data processing can also be achieved on the local GCS. For this discussion, we use the term “data” to refer to any information relevant for operations (control commands sent to the vehicle, diagnostic and situational awareness information coming from the vehicle, and such) along with measurements telemetered from the sensors. Data packets are streamed in real time between the WxUAV and the GCS via MAVLink (Micro Air Vehicle Link) messages. Data packets coming to the ground station are channeled into two streams: one goes to software operating on the local GCS and one goes to a Cloud interface, which forwards the MAVLink messages to an Event Hub in the Cloud, where processes running on scalable virtual machines read and process messages from the Event Hub, perform additional calculations and aggregate MAVLink messages into a single data record. Real-time processing and visualization can also be achieved directly on the ground station computer. Data records sent to the Cloud contain all variables for a single WxUAS at a single point in time. These records are persisted in a NoSQL database and forwarded to a Service Bus for publication to connected subscribers. Additionally, flight commands can be sent from designated remote subscribers (controllers) to ground computer, which are relayed to the WxUAS vehicle. This is illustrated in Figure 11.

A critical role of the data processing architecture will be that of monitoring and managing flight operations. It was mentioned earlier that information from the GeoFence Radar and the ADS-B system will be used to deconflict the air space; however, it was not mentioned how this will be accomplished. For expediency and safety, the processing will be largely conducted on the WxUAV and GCS. That is, the GCS will monitor the data streams from the risk-mitigation instruments, e.g., GeoFence Radar and ADS-B system, then assess whether or not a flight can be initiated or should be aborted. If the WxUAV is in flight, then the GCS will communicate with the WxUAV and plan evasive maneuvers to avoid in-air collision. This command-and-control interface is still in development.

The data flow depicted in Figure 11 only goes from the WxUAV to the GCS and then to the Cloud; however, we are developing a custom communication framework to allow for two-way data flow. MAVLink and MissionPlanner already support two-way data flow and this is part of our current operations. However, we must implement and utilize more customization and flexibility for unattended operations than is commonly needed when the WxUAV operator is on site. Moreover, we may find it necessary to evolve beyond MissionPlanner as an autopilot tool.

The success of the 3D Mesonet will not only depend on our ability to provide raw data to models but also to render data visualization products. The data display should be versatile and able to manage data streams from several WxAUVs and other data feeds. For this task, we can share computation burden with our cloud-based computing resources. Remote users may opt to visualize data streams differently, depending on their particular interests. Thus, the data visualization software should be versatile and configurable.

### 5.2. Data Examples

Here, we provide examples of measurements collected using a single or a few WxUAS. Logistically, it is challenging to sample the atmosphere at multiple, spatially separated locations because of personnel issues. This is why it is important to move towards unattended operations. However, even data collected at a few sites provides valuable information pertaining to the thermodynamic and kinematic state of the ABL. Data from the campaigns discussed below are still being evaluated, but the results provided reveal the value of using WxUAS to sample the ABL. There have been some reports in the literature of using combined rotary-wing and fixed-wing WxUAS to obtain coordinated measurements in the ABL [18,37,46]. We focus on observations from the CopterSonde operating in a profiling mode under a wide range of atmospheric conditions.

The first robust field testing of the CopterSonde in approximately its current configuration occurred in February 2018. A team of researchers from five universities descended on a remote island on the Finnish side of the Bay of Bothnia to study the stable Arctic atmospheric boundary layer. The University of Oklahoma CASS team was invited to join the Finnish Meteorological Institute (FMI), The Geophysical Institute of the University of Bergen (UiB), University of Tübingen, and the University of Applied Science Ostwestfalen-Lippe (OWL) for ISOBAR, or Innovative Strategies for Observations in the Arctic Atmospheric Boundary Layer. The four-week sampling combined more conventional ground-based instrumentation such as sodars, lidar, flux stations, and sonic anemometers with small WxUAS to characterize the stable boundary layer in Arctic-like conditions. In addition to completing the scientific goals of characterizing the stable boundary layer, the CASS team achieved many firsts in operations using the CopterSonde, such as flying at night and beyond visual line of sight, and setting a new altitude record by climbing to 1800 m (5900 ft) AGL. The CASS group participated in several intensive operational periods in conjunction with the other teams to collect continuous boundary layer profiles over the course of 16–24 h. This provided a means to observe the diurnal evolution of the stable boundary layer that would have been impossible by one crew alone.

In July 2018, the CopterSonde was deployed to the San Luis Valley in south-central Colorado for the Lower Atmospheric Process Studies at Elevation—a Remotely-piloted Aircraft Team Experiment (LAPSE-RATE). LAPSE-RATE was organized in conjunction with the International Society for Atmospheric Research using Remotely-piloted Aircraft (ISARRA) meeting in Boulder, CO. During the six-day field campaign (14–19 July 2018), over 100 participants from 13 institutions participated in LAPSE-RATE. Collectively, 35 WxUAS were deployed to collect data over 260 flight hours distributed across 1287 flights. The scientific objectives can be broadly characterized as studying environmental conditions leading up to convective initiation, studying drainage flows originating from the surrounding mountains, and characterizing conditions in the ABL during the morning transition period. Another major objective of the campaign was to validate measurements taken from platforms flown by the various universities and industry partners [40].

**Figure 12:**
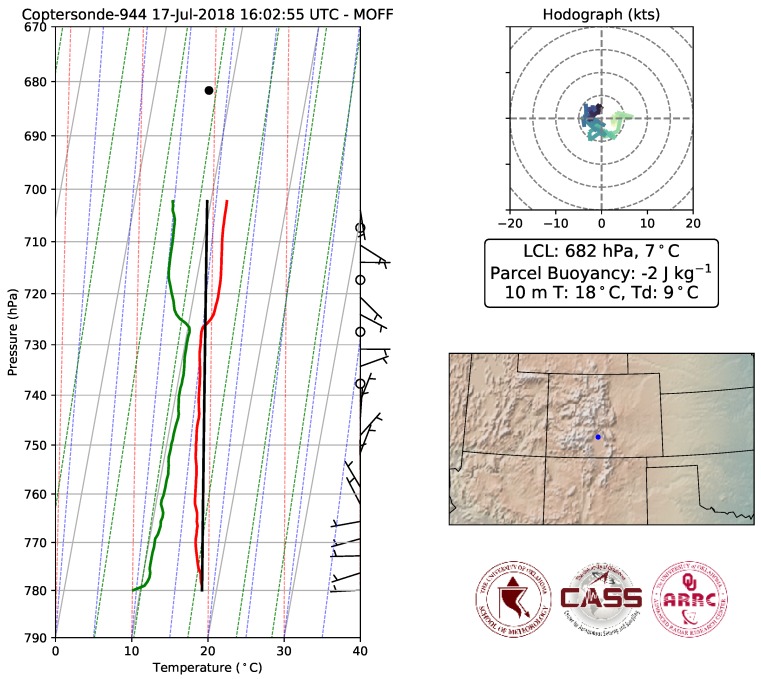
Example of data collected on 17 July 2018 in Moffat, Colorado using the CopterSonde as part of the LAPSE-RATE field campaign. The data correspond to an early morning transition of the ABL. The leftmost panel shows temperature (red line), dewpoint temperature (green line) and the calculated parcel trajectory (black line) plotted on a skew-T log-p thermodynamic diagram. The black dot indicates the calculated lifting condensation level (pressure: 682 hPa, temperature: 7 °C). Wind barbs, expressed in knots, are also displayed. The map indicates the location of the measurements. The upper right plot is a hodograph of the winds expressed in knots. Temperature and dewpoint temperature near the surface at 10 m were 18 °C and 9 °C, respectively.

During the LAPSE-RATE, researchers from the University of Oklahoma were positioned at two locations within the valley: the Moffat, CO Consolidated School and the Saguache, CO Municipal Airport. Data from one of the flights at the Moffat School is shown in Figure 12. These data were collected as part of an intensive observation period to observe the morning transition. Flight permissions allowed WxUAV sampling us to 3000 ft (910 m) AGL. The data shown in Figure 12 were collected around 10:00 local time after the surface inversion had mixed out. The top of the boundary layer can be seen at a pressure level of approximately 727 hPa.

**Figure 13:**
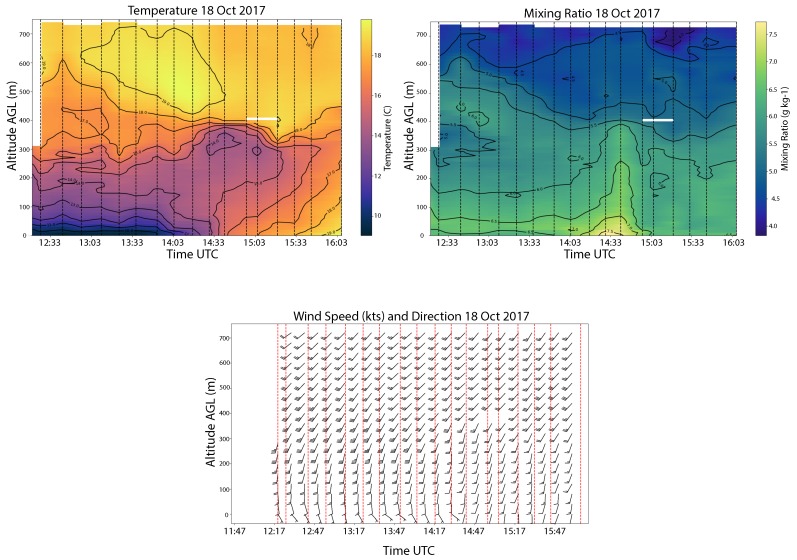
Time-height profiles of temperature, water vapor mixing ratio, and wind from data collected on 18 October 2018 at KAEFS in Oklahoma using the CopterSonde. Eighteen profiles were collected. The vertical dashed lines in the upper two plots and vertical red lines in the lower plot indicate the times of the WxUAS measurements. An interpolation scheme was used to generate the time height plots shown. The data correspond to a boundary layer transition from stable to well mixed. There was a vertical surge of moisture at the time of the transition.

Numerous deployments have also taken place just south of the University of Oklahoma campus at KAEFS. An example of data collected during a complicated morning transition at KAEFS is shown in Figure 13. These data show many interesting features. Flights started at civil twilight and went through the full morning transition. The temperature plot shows a strong nocturnal inversion (Figure 13, temperature) creating a stable boundary layer from the surface to 800 m and a residual layer above that (see Figure 1, profile A). As the sun continued to rise, the surface warmed rapidly between 14:00 UTC and 15:00 UTC, creating a multi-layered profile (Figure 1, profile B). At 14:30 UTC, a moisture surge was observed (Figure 13, mixing ratio) that was coincident with observed evaporation of dew from the grass. After this surge, the moisture mixed throughout the profile and became well mixed in height. Finally, dissipation of a nocturnal low-level jet (LLJ) was observed at approximately 400 m (Figure 13, wind).

For some deployments at KAEFS, the Collaborative Lower Atmospheric Mobile Profiling System (CLAMPS [47]) was co-located with WxUAS operations. CLAMPS is a system designed to fill the same gap WxUAS is intended to fill and utilizes three main instruments: a scanning Doppler lidar (DL), an Atmospheric Emitted Radiance Interferometer (AERI), and a microwave radiometer (MWR). The DL is capable of measuring profiles of wind speed and direction while the AERI and MWR are able to retrieve profiles of temperature and moisture. In addition to the remote sensors, CLAMPS is equipped with a Vaisala radiosonde Sounding System and is able to store helium tanks for radiosonde launches.

**Figure 14:**
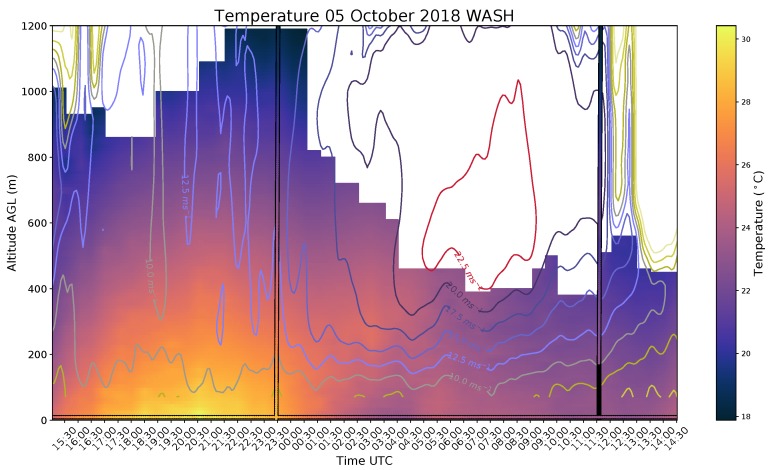
Time-height profiles from data collected using a profiling WxUAS, CLAMPS, and radiosondes. The background shaded contour corresponds to temperature data from the CopterSonde. The labeled line contours represent wind speed derived from the DL. The two color-shaded vertical columns and the single color-shaded horizontal line at 10 m represent temperature measurements from radiosondes and from the Washington, Oklahoma Mesonet site (located at KAEFS), respectively.

Figure 14 shows data from a handful of instruments that were deployed at KAEFS. These data are from an in-house campaign, known as Flux Capacitor, in October 2018 to evaluate the CopterSonde performance over a full diurnal cycle. During this campaign, OU operated under a Certificate of Authorization that allowed flights to 5000 ft (1520 m) AGL during the day and 4000 ft (1220 m) AGL overnight. However, line of site to the CopterSonde is usually unobtainable past 4000 ft AGL. Flights were performed every 30 min for the full period.

The combination of CopterSonde and CLAMPS data helps shed light on features present in the 10 m temperature from the Washington, OK Mesonet site. After sunset, the expected radiational cooling at the surface was present from 00:00 UTC to 04:00 UTC. After 0400 UTC, warming and an increase in wind speed (not shown) was observed at the surface. Around this time, the DL observed a robust low-level jet (LLJ) form. The strength of the winds in the LLJ limited the max height of the UAS, thus observations were limited to heights below the LLJ core. However, the CopterSonde observed what was likely downward mixing of warm air and momentum, which caused the warming and wind speed increase at the surface. This would be unobservable without the WxUAS and DL since the entire event occurred between the typical radiosonde launches.

## 6. Conclusions and Future Directions

As we have noted, there is a clear need to improve our understanding of the Earth’s ABL, a view that appears frequently in several national studies [4,5,6,7,8]. However, due to the complexity and temporal variability of structures in the atmospheric boundary layer, most conventional atmospheric sensing methodologies, such as radiosondes, weather radar, ground-based remote sensing instruments and satellites, fall short of providing the spatial and temporal resolution needed to understand boundary layer processes.

The continuing deployment of small WxUAS to collect in situ measurements of the atmospheric state in conjunction with surface conditions will significantly expand weather observation capabilities. Having the ability to deploy networks of WxUAS could significantly enhance the safety of individuals and support commerce through improved observations and short-term forecasts of the weather and other environmental variables in the lower atmosphere. In particular, observations from a 3D Mesonet could play a critical role in helping to address several pressing scientific questions, including several outlined in the most recent National Academies’ Decadal Survey “Thriving on Our Changing Planet: A Decadal Strategy for Earth Observation from Space” [6]. Examining the list of “most important” questions identified in the report, we find several, which could be better addressed through the availability of improved observations of the ABL, including: How do processes in the ABL relate to air–surface (land, ocean, and sea ice) coupling? What drives the timing and location of convective storms and heavy precipitation? What processes are most relevant in determining the spatiotemporal distribution of air pollutants? How is the water cycle changing and what impact does that have on droughts and pluvials? How are the energy and water cycles being affected by anthropogenic effects? Although taken from a report with a focus on Earth observations from space, they are motivated by the need to improve measurements in the lower atmosphere, regardless of methodology.

Clearly a novel approach for ABL monitoring such as WxUAS must be developed and tested to fill this data gap. Moreover, we recognize that WxUAS technology is not a panacea. Rather, it is simply a *means to an end*, with the *end* being a better understanding of of the Earth’s atmosphere and its processes. Therefore, a holistic approach should be envisioned that involves complementary observational data from a variety of sources. Additionally, carefully constructed modeling approaches should be adopted to best utilize the observational data. This has been our guiding philosophy when developing the 3D Mesonet concept.

As outlined in this discussion, we have been able to conceptualize, develop, and successfully test many of the individual components needed to deploy a 3D Mesonet station; however, there is still much to be done before completing a fully autonomous prototype system. For example, we must identify a suitable charging station for the WxUAV based on several design considerations. The WxUAV must be able to execute a precision landing on the charging station. There must be some mechanism for aligning the aircraft with an inductive or direct connection charging port or to physically swap the battery of the WxUAV. The charging station and the WxUAV should be protected from the elements when not in use. There are commercially available rotary-wing UAS, which offer autonomous charging capability; however, we must find or build one suitable to our particular aircraft and sensor suite.

Another critical consideration when designing a 3D Mesonet prototype is that of system integration. For example, the GeoFence Radar must have software in place to reliably detect and track manned aircraft. Furthermore, when it has been determined that the aircraft has entered into geofenced air space, appropriate deconfliction actions must be taken, which could involve a variety of actions depending on conditions: delay launch, return to launch area, create separation between the manned aircraft and the WxUAV, and so forth. Similar actions could be taken if a manned aircraft is detected through its ADS-B out signal. Creating system software to allow for safe and reliable operations will require considerable development and testing.

With these considerations in mind, we continue to work towards developing a prototype of a 3D Mesonet WxUAS station, which can operate unattended. In addition to the hardware, software, sensor, and system engineering challenges, we realize that there are regulatory hurdles to overcome before a 3D Mesonet can be implemented. As previously mentioned, several risk mitigation factors are already developed or in development. Additionally, we must consider the ability of the system to operate in a range of environmental conditions, including: extreme temperatures; strong winds and turbulence; and icing. The current limitations of the system are being explored. Hardening the WxUAS against adverse weather conditions is a topic of ongoing research. During field campaigns, the CopterSonde itself has been operated in a temperature range of −25° to 30°, in winds up to 25 m s${}^{-1}$, to altitudes of 10,000 ft (3050 m) MSL, and in clouds.

As WxUAS technology continues to mature and our capacity to make robust and accurate observations of the lower atmosphere grows, we must correspondingly match this development within the realm of atmospheric modeling. Once data are available from WxUAS deployments, these observations can be assimilated into NWP models along with all other available weather data to determine the extent of improvement to the model forecasts and the longevity of the impact with a focus on high impact weather events depending on the season and location. These types of modeling studies are known as Observing Simulation Experiments (OSEs) [48,49]. The University of Oklahoma’s Center for Analysis of Storms (CAPS) has experience using a number of NWP models, including ARPS, WRF and the designated next-generation global prediction system (FV3), including performing mesoscale OSEs [50]. Data assimilation can be accomplished for in situ data such as the WxUAS with efficient methods such as 3DVAR analyses assimilated with Incremental Analysis Updating [51], recently updated at CAPS to include variable-dependent timing [52] as well as more complex ensemble-based methods such as Square-Root Ensemble Kalman Filter (EnKF).

At this stage, the 3D Mesonet concept is only a dream, but from dreams, reality can happen. Such a network would carry us closer to filling the atmospheric data gap that exists in the ABL and help us to better understand complex surface–atmosphere interactions and energy exchanges. Admittedly, as we move forward, we must also be open to other complementary and evolving sampling technologies to collect these data. Currently, however, WxUAS offer much potential. The needs of society are placing ever increasing demands on forecasters to improve the granularity and fidelity of weather predictions. This in turn requires networks of atmospheric observing networks along with the ability to integrate the observations efficiently into NWP models. There is work to be done to get there. We feel that realizing the dream of a 3D Mesonet can play a critical role in reaching these goals.

## Figures and Tables

**Table 1:** Desired Meteorological Measurement Specifications for the CopterSonde.

**Meteorological Variables and Accuracies**	
Temperature	±0.2 °C
Relative Humidity	±5.0%
Pressure	±1.0 hPa
Wind Speed	±0.5 m·s${}^{-1}$
Wind Direction	±5 Degrees Azimuth
**Sensor Response Time**	
Time	<5 s (Preferably <1 s)
**Operational Environmental Conditions**	
Temperature	−30 to 40 °C
Relative Humidity	0–100%
Wind Speed	0–35 m·s${}^{-1}$

**Table 2:** Specifications for the uAvionics PingStation.

Specification	Value
Input Voltage/Power	44–57 V/500 mW (Power over Ethernet)
Size	4.75” × 2.0 ” × 3.25” (box) 9.5” (antenna)
Weight	340 g
MTL 1090 MHz	−88 dBm
Dynamic Range	−79 to 0 dBm
MTL 978 MHz	−93 dBm
Dynamic Range	−90 to −3 dBm
